# To be present, share and nurture: a lifeworld phenomenological study of relatives’ participation in the suicidal person’s recovery

**DOI:** 10.1080/17482631.2017.1287985

**Published:** 2017-02-28

**Authors:** Linda Sellin, Margareta Asp, Tomas Kumlin, Tuula Wallsten, Lena Wiklund Gustin

**Affiliations:** ^a^ School of Health, Care and Social Welfare, Mälardalen University, Västerås, Sweden; ^b^ School of Health, Care and Social Welfare, Mälardalen University, Eskilstuna, Sweden; ^c^ Centre for Clinical Research, Uppsala University, County Hospital, Västerås, Sweden; ^d^ Department of Health and Care Sciences, UiT/The Archtic University of Norway, Campus Narvik, Norway

**Keywords:** Caring science, interpersonal relationships, lived body, mental health nursing, person-centred care, phenomenology, psychiatry, relatives’ experiences, suicidality, vitality

## Abstract

In today’s health care, participation is acknowledged as important. However, there is limited research on how relatives of patients at risk of suicide experience their opportunities to participate in care during periods when their close ones are subject to inpatient care. The aim of this study was to describe the phenomenon of participation, as experienced by relatives of persons who are subject to inpatient psychiatric care due to a risk of suicide. The study was conducted through a reflective lifeworld research (RLR) approach, based on phenomenological philosophy. Eight relatives of patients receiving care from professionals in a psychiatric specialist health care context in Sweden participated in phenomenon-oriented interviews. Data were analysed to elucidate a meaning structure of the phenomenon. The findings show that the phenomenon of participation was more associated with patients’ recovery processes than with the caring process, and means “being actively involved in a process in which the person regains the desire to live”. The meaning of participation is further described by its meaning constituents: struggling for being able to be present for the person at risk of suicide, being able to share everyday life, and nurturing sources for vitality. These insights into the meaning of participation highlight the importance of allowing supportive relatives to be a part of the patient’s life, while the person is cared for in an inpatient hospital setting. Thus, participation enables relatives to be acknowledged as resourceful human beings in the patient’s recovery process, and thereby facilitates a sense of being able to manage and share life itself together with the person. This means that mental health nurses need to recognize individual variations of relatives’ participation processes, and take on the responsibility of acknowledging relatives’ lifeworlds.

The World Health Organization (WHO, 2014) highlights that one person dies of suicide every 40 s somewhere in the world. This requires strategies that have the potential to manage the challenges of preventing suicide in a profound way. Even though experiences of loneliness are a risk factor for suicide (Levi-Belz, Gvion, Horesh, Apter, 2013; Nagra, Lin, & Upthegrove, 2016), many people who are considering suicide as a way out have significant others, such as family and/or friends. Hence, people in the suicidal person’s social networks have an important role in suicide prevention, and can experience challenges through the burden suicidal behaviour places on the family (Owens et al., 2011; Sweeney, Owens, & Malone, 2014). Sometimes these challenges are related to stigmatizing attitudes and a lack of knowledge regarding suicide, which can lead to obstacles for support (Peters, Cunningham, Murphy, & Jackson, 2016). Thus, relatives are a resource for the suicidal person and can contribute knowledge from their perspective of the situation, but also need support to be able to handle the situation and give support to the person.

Mental illness is a common risk factor for suicide (Lönnqvist, 2009). Thus, health-care research is dominated by discussions that focus on understanding suicide in the context of mental illness (Cutcliffe & Stevenson, 2008; Roberts & Lamont, 2014). Research considering patients’ perspectives reinforces the idea that it is not enough to only use a traditional view of suicide, as a focus on understanding suicide in the context of mental illness can hinder an attitude of openness in the professional caregivers’ responses to the suicidal person (Lakeman & FitzGerald, 2008; Vatne & Nåden, 2014). In addition to research that emphasizes the suicidal patient’s own perspective, research should also recognize relatives, as experience of being connected to others is necessary for life (Vatne & Nåden, 2016). Special focus should be on engagement about how to build trust and meeting specific needs in encounters with suicidal patients and their significant others (Gaebel et al., 2014). When suicidal persons receive psychiatric care, mental health nurses should support relatives as a resource for the patient (Barker & Buchanan-Barker, 2011; Cutcliffe, Stevenson, Jackson, & Smith, 2006; Talseth & Gilje, 2011). However, research on how relatives can be supported to contribute in the care of the suicidal person is limited (Chiang, Lu, Lin, Lin, & Sun, 2015; Sun, Chiang, Yu, & Lin, 2013), compared with research considering the role of professional caregivers in identifying and managing risk to self (Hawton, 2016; Herron, Ticehurst, Appleby, Perry, & Cordingley, 2001). There is some research considering relatives’ experiences of participation in the care suicidal family members receive from psychiatric inpatient care. Talseth, Gilje, and Norberg (2001) describe that relatives’ experience of being met in a humanizing relation with professional caregivers may create hope for relatives. In contrast, experience of being excluded from the care and left alone may increase the relatives’ experience of taking responsibility for the suicidal person who is outside their control. Available and supportive relatives in the suicidal person’s social networks may be key persons for the patient. Relatives have described their engagements, but also their despairs, while striving for professional help for the person (Talseth et al., 2001).

Even though relatives are considered to be important resources in the care, it is unclear what challenges they are faced with and what support they need to participate in a meaningful way. Owens et al. (2011) highlight that their proximity to the suicidal person and their emotional investment in the relationship can make it difficult for them to interpret signs of suicidality and to know how to act. Hence, strategies to strengthen the capacity of significant others to intervene in preventing suicide are needed and should acknowledge these difficulties. Owens et al.’s (2011) study, as well as studies that highlight aspects of participation in other contexts (Björk Brämberg, Nyström, & Dahlberg, 2010; Frank, Asp, & Dahlberg, 2009; Todres, Galvin, & Dahlberg, 2014), give rise to questions about participation as something that exceeds a view of participation as decision-making in care plans. This calls for research that accounts for what participation means for the relatives themselves. Therefore, the aim of this study was to describe the phenomenon of participation, as experienced by relatives of persons who are subject to inpatient psychiatric care due to a risk of suicide.

## Methodological approach

This study was carried out under the guidance of a reflective lifeworld research (RLR) approach, as described by Dahlberg, Dahlberg, and Nyström (2008). The “lifeworld” is a world of meaning, in which individuals experience and share the world in relation with other humans. Human beings reach this personal and “intersubjective” world through the “lived body”. This lived world and body is embedded in coherence of “coexistence,” and manifests itself through human “lived experiences” (Merleau-Ponty, 2013/1945). This philosophical point of view considering human beings as embodied subjects, who have their being in the world, is not a matter of being an object acted upon by external forces. It is rather a matter of having certain active ways of interacting with the world. The mode of existence we have is “being-in-the-world”, in which we experience and describe the world from our own subjective point of view (Bengtsson, 2013; Merleau-Ponty, 2013/1945). Thus, this ontological and epistemological knowledge contributed to foundations for the methodological practice in this study. The notion of the lifeworld and the lived body shed light on the opportunities to explore the phenomenon in focus from the persons’ own lived experiences. This research approach acknowledges that phenomena in the world such as participation, are subjectively experienced by individuals, and there is also a common thread of meaning abstracted and described as the “essence” (Dahlberg, 2006). This essence makes the phenomenon into what it is, and includes the variances of the essential meaning, the so called “meaning constituents”. This unity of the essence and the meaning constituents, describes the phenomenon’s “meaning structure”. In order to apply this research approach, the researchers need to embody a “phenomenological attitude”, in which “openness and sensitivity” to the complexities of the lived experiences are established. This involves the process of “bridling” (Dahlberg et al., 2008) the researchers’ understanding and also reflection on the meanings. The concept of bridling includes slowing down the process of understanding as a whole, which includes restraining one’s pre-understanding, to avoid making definite what is indefinite in human existence as being-in-the-world with others (Dahlberg & Dahlberg, 2003, 2004).

### Participants and setting

Participants were recruited from the relatives of participants from a previous study (Sellin, Asp, Wallsten, & Wiklund Gustin, 2016). The first author asked participants in that study to invite relatives that they considered as important persons in their life. If the persons agreed, they were contacted by the first author and given further information about the study. The inclusion criteria for this study were that participants: (1) were identified as an important person by a patient who was admitted to psychiatric inpatient care related to suicide risk, defined by a clinical suicide risk assessment, made by psychiatrists at the psychiatric clinic; (2) were at least 18 years old; (3) were able to understand and speak the Swedish language; and that (4) the interview could be conducted within 4 weeks of the patient’s admission. Five women and three men, aged between 30 and 80, were included in the study. They were all close relatives to the patients.

### Data collection

The place for the interviews was chosen according to participants’ wishes, to enable them to feel comfortable and at home in a neutral place. Six phenomenon-oriented interviews (Dahlberg & Dahlberg, 2003, 2004; Dahlberg et al., 2008) were carried out in conversation rooms at a health care or a university setting. One participant chose a telephone interview and another participant chose to be interviewed at home. All interviews were conducted by the first author. With respect to the challenge that lived experiences can be difficult to narrate, the interviewer was attentive to the verbal and nonverbal communication of participants. In so doing, the interviewer supported the narrative by being sensitive to the story, and also by being present and showing respect to the participant (Wiklund-Gustin, 2010). The initial question encouraged participants to describe their experience of participation while their close relatives were cared for in a psychiatric inpatient setting. In order to support participants to elaborate their descriptions, follow up questions were included such as: “Can you tell me more about that?”, “What does this mean for you?” All participants were encouraged to talk about what mattered from their own perspectives of the phenomenon, with regard to their own subjective experiences. In this way, the interviewer strived to bridle her understanding, and remain open and sensitive to both the participants and the phenomenon itself. Participants’ personal descriptions of their experiences provided variation in the data. The eight interviews lasted between 45 and 119 minutes, and were recorded with a digital voice recorder, and transcribed verbatim.

### Ethical considerations

The study was approved by an ethical review board (registration number 2013/123–3/4), and conforms to the ethical principles outlined in the Declaration of Helsinki (World Medical Association, 2008). The research was conducted with respect and responsibility for confidentiality, and protected the participants’ integrity and identity. Considering the risk that sharing one’s experiences in an interview could arouse distressing thoughts for the participants, an offer to contact the interviewer afterwards was made. All participants were also given oral information that they could turn to the psychiatric professional network if the interview raised issues that required a follow-up conversation. Written informed consent was obtained from all participants before the interview. All participants were given opportunities to withdraw or to pause the interview at any time if they wanted to (Liamputtong, 2007).

### Data analysis

The methodological principles (Dahlberg, 2006; Dahlberg & Dahlberg, 2003, 2004; Dahlberg et al., 2008) carried out in this study involve a dialogue between the researchers and the transcribed text, in order to illuminate and describe the phenomenon’s meaning structure. The text was read repeatedly while listening to the recorded interviews to reach an understanding of the contents as a whole. Continuing this phenomenological attitude of openness and sensitivity to the text as a whole, the text was further analysed to uncover particular nuances of meaning. Meaning units relevant to the aim of the study were identified and transformed. The meaning units were then compared, to identify differences and similarities. The meaning units that were considered to be similar, or related in other way, were grouped into clusters as a preliminary analysing stage, on the way to elucidate a meaning structure of the phenomenon of participation. The clusters were then approached through discussion and reflection, in order to understand the essential meaning of the phenomenon. In the reflection at this level, “bridling” the researchers’ understanding involved working through the emerging meanings, and understanding each meaning as a figure against the background of the others. The bridling allowed the researchers to slow down the process of understanding the phenomenon, and in this way, await its meanings to show themselves. This included at the same time maintaining sensitivity in the continuing process of discovery. When the meanings relevant to the phenomenon had been identified, and no inconsistency could be found, the essence of the phenomenon was expressed and described. As a final stage, the meaning constituents that were considered as the variances of the essential meaning were formulated.

The meaning structure of the phenomenon of participation as experienced by relatives of persons who are subject to inpatient psychiatric care due to a risk of suicide is presented below. The description of the meaning structure includes both the essence and its meaning constituents (Dahlberg et al., 2008). The use of quotations intends to illustrate the participants’ lived experiences. In order to increase readability and with regard to the context in focus, the concepts “person”, “loved person” and “family member” are used instead of “person at risk of suicide”, i.e., the patient.

## Findings

The essence of the phenomenon of participation as experienced by relatives of persons who are subject to inpatient psychiatric care due to a risk of suicide, means “being actively involved in the process in which the person regains the desire to live”. Being actively involved entails an engagement to influence the situation, with regard to the person’s wishes and needs. It is about standing up for one’s experiences as a valued relative, and reaching through towards participation. Being actively involved is facilitated by a mutual sensitivity between the persons who are connected. Being included in such a connection can reinforce a sense of shared understanding in everyday life. This is supported when the relatives gain knowledge about the present situation, as ways to face what is going on. Being actively involved in this sense also means being deeply touched by the person who struggles with suicidality. Experience of not being able to understand what is happening can arouse experiences of being disconnected from what is going on. Struggling for participation in such ways entails a fear of losing the loved person which one cares about and may feel unable to help. Hence, experience of being disconnected from what is going on can appear as a threat to existence, which can take resources from one’s ability to be present as a worthy relative. Being able to carry through the relation with the person in accordance with the other’s wishes and needs nurtures the experience of being actively involved. Participation is also reinforced by acknowledging one’s own worth as a relative and awareness of the otherness in one’s experiences. The meaning of participation is further described by its meaning constituents: struggling for being able to be present for the person at risk of suicide, being able to share everyday life, and nurturing sources for vitality.

### Struggling for being able to be present for the person at risk of suicide

This aspect of participation means being present as a helpful resource. Experience of being able to be present as a helpful resource for the loved person is crucial when the person is struggling with suicidality. This resourcefulness contributes to relatives’ experience of bearing in the concrete situation. This includes creating a space for connectedness with the persons who are concerned, and taking into account the value of being present for the loved person. One of the relatives described her experience:

I believe that the times I have really been able to be there, have given a lot, because no one wants that someone in the family should feel excluded. I mean, she feels when we don’t bear anymore (…) I believe that the time when you really were able to be there, and she [the sister] could turn to me and talk, has given a lot.

Reaching one’s own resourcefulness can be challenging, as awareness of the risk of losing the person can involve doubt about one’s ability to contribute in a meaningful way. The doubt evolves by experience of lacking knowledge about the situation, and can take resources from one’s experience of being a worthy relative. Being able to understand the situation facilitates being present with regard to the loved person’s concerns. Being able to take steps towards such presence is also facilitated when professional caregivers invite relatives to be included in the concrete situation. The following quote illustrates one of the relative’s experiences when her husband is cared for in an inpatient hospital setting:

And one time when I came to visit after dinner, we met a professional caregiver who was great. And she said “we are going out on the front side to smoke, do you want to join us?” And I just said “oh yes.” And the other patients that were in the company said that it was okay, and it became a great time. I just joined them and it wasn’t so complicated. (…) it made it more natural (…) we talked about things like the sun had come early and such ordinary things. (…) and that she asked the other patients, otherwise I wouldn’t probably have joined them.

Thus, struggling for being able to be present for the person means reinforcing yourself as a valued relative, and simultaneously questioning one’s ability to contribute in a meaningful way. The doubt and the questioning are balanced by experiences of connectedness with the persons who are concerned. This connectedness, in which relatives are allowed to take into account the value of having links with the persons who are concerned, nourishes relatives’ feelings of bearing in the concrete situation.

I get a little more energy because it was a difficult autumn that ended in a suicide attempt, but I have actually been able to let that go. Not all, but after this turn he [the husband] was offered contact with a nurse which he agreed to. I think it was every 14th day and it feels good that I am not all alone in it.

Experience of being included in this connectedness facilitates relatives to be present and respond to the other in a way that supports participation. Being actively involved in such ways is experienced as vital for relatives in the process in which the person regains the desire to live. This is further facilitated in an experience of closeness with the person.

I believe it has probably… it gives a lot when the family manages it and when you bear to be close, and you bear to be there. Thus, it is enough just to sit and watch TV together, just such a thing. Just that you are demonstrating that I am here. So that means probably a lot I believe.

Struggling for being able to be present for the person at risk of suicide means facing that life is vulnerable and not something that can be taken for granted. This awareness of life’s circumstances reinforces one’s engagement to be present as a worthy relative. Reaching one’s own resourcefulness in connectedness with the loved person facilitates participation as it means taking into account what matters in everyday life.

### Being able to share everyday life

This aspect of participation means that relatives search for contact with the person at risk of suicide, as a way of being able to participate in what is going on. This encompasses asking questions to the person, which can facilitate the other to express what is going on. Searching for contact in such ways can facilitate understanding of how to carry out the situation with regard to the loved person’s s wishes and needs. This search for contact includes a need for reciprocal communication with the professional caregivers. When this communication is available with the professional caregivers, openness in the contact facilitates experience of being actively involved in the concrete situation. One relative described her experience of calling the psychiatric clinic: “When I called I got an answer. I got an answer because she [the daughter] had approved it. And then I feel calmer at once, okay then I know that. And if something happens, then they will call me.” Being able to share everyday life is particularly important when the suicidal person temporarily loses the ability to put words to one’s experiences, and needs a relative who can contribute in one’s case. This means that there are situations where relatives need to contribute in the concrete situation, in order to feel safe and secure about what is going on in relation to the person.

(…) I have been on some of these meetings, a meeting for the week, but it is on her terms, when she has felt offended by the doctors who are going to have their meetings. Then she has wanted support from her Mum or Dad or both. And then you have been supporting her and talked with them.

This means that participation is rather about supporting your family member than joining forces with the professional caregivers. Supporting your family member and being able to find solutions that all can agree on facilitates the experience of being able to influence the situation in a meaningful way. On the other hand, when openness is lacking, and it is not possible to get contact with professional caregivers, this can evoke an experience of powerlessness that affects one’s ability to reach through. Thus, struggling for participation means that relatives of patients at risk of suicide are faced with challenges in their concern to be included and share what is going on. Being able to share everyday life also means dealing with uncertainty concerning what the loved person’s wishes and needs are. This includes holding back one’s own needs, and adjusting to the other, as a way of creating space where the person can be supported in his/her struggle between life and death. Hence, being able to share everyday life is a question of maintaining contact when the person is emotionally distant. This focus on the other’s everyday life is experienced as crucial when the person is at risk of suicide, as it facilitates responding to the other in a meaningful way. The following quote illustrates one of the relative’s experiences when his daughter is cared for in an inpatient hospital setting:

(…) she is afraid of sleeping and gets anxiety when she goes to bed, and it is then she cuts herself during nights with a razor blade. And she was affected by these problems even now during inpatient care (…) so she wasn’t allowed to go out by herself in the beginning, but then, the last week, weeks she was allowed to do so. (…) then she calls me and says that she is at the shopping centre, and that they have razor blades in the store. “Go away from there” I said. “No, I can’t.” I was on my way home from work then (…) so I turned back and drove her to the hospital so she wouldn’t buy. (…) so we are dealing with these problems when they come back.

To participate in what is going on is an expression of relatives’ desire to be available, and to talk about and take part in the family member’s life. This space for connecting and sharing in everyday life is a resource for the persons who are concerned, and needs to be acknowledged in the process in which the person regains the desire to live.

### Nurturing sources for vitality

Being a relative to a person at risk of suicide means dealing with one’s awareness of the risk of losing the loved person. This also means facing questions about life and death in relation with the person. Nurturing sources for vitality can thus be understood as ways of holding on to possibilities of life, with regard to the other’s concerns. These expressions of engagement are grounded in a will to contribute to the loved person’s survival. Nurturing sources for vitality facilitates encouraging expectations of some meaningful change towards the future.

The only thing I can say is that “it will be better.” But how can I know that? I haven’t experienced that, I don’t know that. I just want to give her [the sister] hope that it will be better. But I don’t know when… maybe no one knows that but… you should be able to give a pointer on the road, showing that it will come.

Participation as nurturing sources for vitality means holding onto the relationship with the suicidal person, and is understood as an expression of caring. This caring can contribute to a context for possibilities of life, while simultaneously agonizing over the risk of losing the loved person. One relative described: “I try to put my energy where I can have an influence, spend time with her [the daughter] when it’s possible and I try to do it as well as possible, and be available and so on.” This relative also expressed: “One day she may be gone a few hours and have committed suicide… (…) it’s almost as if we are thinking that it will happen.” This caring for the other nourishes the ground for one’s being and relation with the person, which facilitates participation in a way that may balance one’s experience of uncertainty and grief. Caring for the loved person also reinforces to acknowledge meaningful alternatives in encounters with the person, as ways of nurturing sources for vitality.

We thought she [the daughter] was so inactive there so it become our way of seeing her and taking the dog with us and taking a walk or going away and having some coffee, because she has some favourites such as the bakery (…) I think we support her at least to do something else, so I experience that this has become a little bit to be our role (…) that we did something else other than meeting each other at the ward and talking.

Nurturing sources for vitality, in which one is engaged to holding onto possibilities of life, facilitates participation by means of one’s will to embrace what is important in encountering with the person. Thus, the power of life towards participation in the loved person’s struggle for continued life is acknowledged, and in such ways a feeling of a possible future is brought into the present. One relative described his experience:

(…) the last times she has tried to commit suicide or… then it often has been me she has taken contact with in some way. So I think that… that we have quite a big importance for… or that we have a very big importance for [the name of the daughter]. (…) When she is going to the social department or something, she often wants someone with her and then it’s often Mum who follows, it’s almost always her who follows… so we try to be close and available (…). And we also have a life with… (…) we spend time with neighbours and things like that, celebrate holidays with different, so we have very… yes established conditions in that way, summer house and things like that.

This nurturing and caring presence provides a foundation for relatives to carry through their relationship with the loved person. Being allowed to take into account the relations to each other in such ways is considered as a resource for being actively involved in the process in which the person regains the desire to live.

## Discussion

The aim of this study was to describe the phenomenon of participation, as experienced by relatives of persons who are subject to inpatient psychiatric care due to a risk of suicide. The findings elucidate that the phenomenon of participation is characterized by “being actively involved in the process in which the person regains the desire to live”. Hence, rather than being related to the delivery of care, participation is about being actively involved in the family member’s process of recovery. Therefore, the findings need to be discussed not only in relation to research that considers participation, but also in relation to recovery. These insights on participation includes the authors’ awareness of that even if relatives are not actively involved in the caring process, professional caregivers need to enable some shared knowledge of what has happened with the suicidal person, for instance cutting, an episode of sincere suicidal ideas and/or plans, or a suicide attempt, as a necessary basis for supporting relatives’ participation.

In the light of Barker and Buchanan-Barker’s (2005) research, recovery can be understood as reclamation of one’s story and one’s life. Hence, family members’ participation could be understood as being actively involved in the process in which the patient reclaims his/her life. This also corresponds with research describing the meaning of recovery from the perspective of patients at risk of suicide, where connectedness with relatives appears to be pivotal for reconnecting with oneself while struggling between life and death (Sellin et al., 2016). In line with Sellin et al.’s (2016), as well as Barker and Buchanan-Barker’s (2005), research, it could be concluded that the elucidated meaning of participation, as being actively involved in recovery, is indeed an interpersonal process. In addition, based on our findings we conclude that in this context what Barker and Buchanan-Barker (2005) describe as “caring with” the patient does not only mean supporting the patient to reach contact with relatives, but also facilitating relatives’ involvement in the person’s recovery. This provides the basis for relatives to acknowledge themselves as worthy and resourceful persons, able to support the loved person and also their understanding of what matters in a common world. In this study, the interpersonal process takes place in different, yet intertwined, aspects of life; it is experienced as related to presence and emotional availability; common actions by being able to share everyday life; and also to participate in joint activities that nurture sources for vitality. Hence, participation is about an active involvement in the present, as it unfolds moment by moment in the process of recovery. Each moment is subjectively experienced, in which the past can come up close and the feeling of the future resonates with experiences through life. Relatives’ concern for the family member’s everyday life expresses an engagement to meet what is in the person’s heart in daily life in the psychiatric hospital, and thereby share what is going on in the person’s life regarding the challenges of one’s recovery and what this means—as described by Dahlberg (2011) and Todres et al. (2014) for one’s “minor and major life projects”. In this study, the major project is understood as life itself, where ability to share everyday life may reinforce conditions for recovery, such as the patient’s reclaiming of one’s story together with others in life. The relational aspect of this participation has been described by Merleau-Ponty (2013/1945) as intersubjectivity. In the encounter between relatives, patients and professional caregivers, intersubjectivity can be a vital source that may support both addressing the patient’s story, and developing a common story with the persons who are connected. Reaching through to each other in these ways may support relatives in enhancing their understanding of their own wishes and needs and also the wishes and needs of the loved person. If nurses are able to strengthen such participation, it can facilitate relatives in carrying through their interpersonal relationships (Curchill, 2012; Todres, Galvin, & Dahlberg, 2007). Thus, participation in the patient’s process of recovery facilitates a sense of being able to manage and share life itself together with the loved person. There is further support for these processes of participation, such as when relatives experience that they have been excluded from the care and left alone, overwhelmed by a feeling of responsibility for the suicidal person (Talseth et al., 2001). Another part of the issue may be ongoing stress like a double trauma, which can include the trauma of the suicide attempt (s) and the following psychosocial impact on the interpersonal relationships in the family (Buus, Caspersen, Hansen, Stenager, & Fleischer, 2013). This corresponds to Sun et al.’s (2013) notion that mental health nurses can provide meaningful caring responses to relatives, by acknowledging their ability to care for their suicidal family member. This should include the opportunity for education for mental health nurses (Gaebel et al., 2014; Gilje & Talseth, 2014; Titelman & Wasserman, 2009), to enhance understanding of the specific needs of relatives, including the ontological and epistemological foundations on which mental health care is based. When doing this, acknowledging individual variations of relatives’ participation is pivotal.

This study also contributes with nuances to the description of person-centred care given by Kristensson Uggla (2014), in which the patient’s narrative is understood as a foundation and prerequisite for the “partnership” between patient, relatives and professional caregivers, where relatives are acknowledged as capable of contributing to the narrative. In this study, partnership is understood as a common project in which the patient, relatives and professional caregivers contribute with their unique competences and exchange experiences: a common project that is possible to carry out for relatives by being actively involved in the process in which the patient regains the desire to live, through the connectedness with the persons who are concerned, in which you are allowed to be a part of the patient’s life. Based on our findings and in line with the contributions of Kristensson Uggla (2014), participation in this context can be understood as being actively involved through recognizing the person both as a suffering and resourceful human being, and also by “nurturing sources for vitality.” This vitality facilitates holding on to the possibilities of life, and means facing questions about both life and death. Thus, participation facilitates relatives being included in what can be understood as a common project to acknowledge meaningful alternatives in dialogue, that helps to express and understand what the current situation means for the persons who are concerned. Thus, dialogue is a certain way of being-with-the-world. The way in which human beings live in the world as articulated by meaning and language is one of the given possibilities in human experience (Bullington, 2013; Merleau-Ponty, 2013/1945). These insights on participation enlighten the importance of a “collaborative approach” (Gysin-Maillart, Schwab, Soravia, Megert, & Michel, 2016) and need to be considered when the intention is to acknowledge relatives as a part of the patient’s life. This also indicates that mental health nurses should take responsibility themselves to reflect on what can support relatives to contribute to the common project in everyday life towards participation. This includes recognizing that, in a situation where relatives are struggling to be able to be present as a resource for the person at risk for suicide, caring for the person can include fear of a repeated suicide attempt and of not being allowed to tell one’s story, and of losing the loved person. If nurses are able to acknowledge revealed sources for vitality, it can have the potential to facilitate relatives’ nurturing and caring presence with the person. Thus, nurses need to embody sensitive understanding of the specific needs of relatives (Gabrielsson, Sävenstedt, & Zingmark, 2014; Galvin & Todres, 2009; Owens et al., 2011).

### Methodological considerations

In this study, using a reflective lifeworld research (RLR) approach (Dahlberg et al., 2008), the question of variation in data is more important than the question of sample size. The collected data are based on a sample with five women and three men that met the inclusion criteria and contributed to variations in data. One aspect of the issue may be that the topic is related to problems difficult to talk about. With respect to the complexities of the phenomenon, the researchers’ adoption of the methodological approach (Dahlberg et al., 2008) guided the interviews as a means of listening to the voice of the participants and at the same time strengthening it. This also means that the interviews were carried out by the interviewer’s awareness of intersubjectivity, in which reflections on feelings of the interviewer as well as the participants are considered as resources for understanding participants’ experiences and the phenomenon (i.e., participation) itself (Dahlberg & Dahlberg, 2003, 2004; Holloway, 2007; Lakeman, McAndrew, MacGabhann, & Warne, 2013). This openness and sensitivity to feelings aimed to take into account the participants and the interviewer as active and living human beings, experiencing the world that they live in (Dahlberg, 2012, 2013). Carrying out bridling (Dahlberg et al., 2008) in these ways in relation to the participants and the phenomenon supported the interviewer in paying attention to the intimate experience of meanings in the present situation (Cavalcante-Schuback, 2006). One particular challenge that needs to be addressed is the balancing between closeness and distance in relation to the participants and what they brought to the interview situation and the phenomenon itself. This shed light on the importance of being respectful to what is bridled, in order to maintain openness and sensitivity towards the other’s otherness (Dahlberg & Dahlberg, 2003, 2004; Todres, Galvin, & Holloway, 2009). This includes awareness that there is always something in the other’s essence that can never be understood (Lévinas, 1990; Todres et al., 2014). Thus, the research approach carried out in this study, in which the dwelling work with meanings is one side of the interviewer’s relation with the participants and the phenomenon, can be seen as one of its main strengths. These aspects of bridling, in which the researchers’ open attitudes, their ability to bridle their understanding and their sensitivity to the phenomenon, are considered to establish objectivity and validity throughout the research. In so doing, objectivity is grounded in openness for understanding the phenomenon in a new way, and being careful to not be too quick to make definite what is indefinite (Dahlberg et al., 2008).

The essential meaning and its meaning constituents contribute to describe certain aspects of the phenomenon of participation. This forms a general understanding (Dahlberg et al., 2008; Hörberg, Ozolins, & Ekebergh, 2011; Lindberg, Österberg, & Hörberg, 2016), where the fruit of application thus hopefully facilitates making visible what has previously not been seen. When applying this knowledge into caring responses in practice, it must take place in sensitive openness to the complexities of human beings’ lifeworlds. This can also be understood with the help of Merleau-Ponty’s (1968/1964) “flesh of the world”, where human beings’ relations with each other and the world imply opportunities to ask questions as ways of further understanding the other’s unique opening to the world.

## Implications for clinical practice and future research

The findings contribute to knowledge about the phenomenon of participation in a process of recovery as experienced by relatives of persons at risk of suicide. A traditional way of understanding participation as decision-making in care plans needs to be complemented with knowledge that describes the meaning of relatives’ experiences of participation. When a person is a relative of a suicidal family member, mental health nurses should support the relative to be actively involved in the process in which the person regains the desire to live. This includes recognizing the person both as a suffering and a resourceful human being in connectedness with others and the world. This connectedness can be a vital source for relatives to nurture a common and reciprocal space of existence with the loved person. Thus, participation enables relatives to be included as resources in the patient’s recovery, and thereby facilitate a sense of being able to manage and share life itself together with the person. This is a common project that is possible to carry out for relatives through the connectedness with the persons concerned, in which you are allowed to be a part of the patient’s life. This is pivotal in psychiatric care where the intention is to be genuinely supportive for relatives of patients at risk of suicide. Thus, mental health nurses need to embody sensitive understanding to the specific needs of relatives, and thereby also be open to individual variations of their participation processes. When relatives are given support to participate in the patient’s recovery, they gain the possibility of sharing experiences at heart of vitality. Further studies that facilitate what participation means would be highly warranted, in order to reach towards more specific caring interventions. The three meaning constituents highlight the value of being present, to share and to nurture as a natural basis for more differentiated studies.
